# A robust synthesis of 7,8-didemethyl-8-hydroxy-5-deazariboflavin

**DOI:** 10.3762/bjoc.12.89

**Published:** 2016-05-06

**Authors:** Matthias Bender, Henrik Mouritsen, Jens Christoffers

**Affiliations:** 1 Institut für Chemie, Universität Oldenburg, Carl von Ossietzky-Str. 9–11, D-26129 Oldenburg, Germany; 2 Institut für Biologie und Umweltwissenschaften, Universität Oldenburg, D-26111 Oldenburg, Germany; 3 Centre for Neurosensory Sciences, University of Oldenburg, D-26111 Oldenburg, Germany

**Keywords:** carbohydrates, heterocyclic compounds, protective groups, riboflavin derivatives, ribose derivatives

## Abstract

The biosynthetic precursor of redox cofactor F420, 7,8-didemethyl-8-hydroxy-5-deazariboflavin, was prepared in four steps from 6-chlorouracil, 2-chloro-4-hydroxybenzaldehyde and bis-isopropylidene protected D-ribose. The latter aldehyde was transformed to the corresponding protected ribitylamine via the oxime, which was submitted to reduction with LiAlH_4_. Key advantage compared to previous syntheses is the utilization of a polyol-protective group which allowed the chromatographic purification of a key-intermediate product providing the target compound with high purity.

## Introduction

The deazariboflavin cofactor F420 plays an important role in bacterial methanogenesis [1–2] and was – in contrast to other, ubiquitous biological redox coenzymes like NAD^+^ or FAD – discovered relatively late (in 1972) [3].

**Scheme 1:**
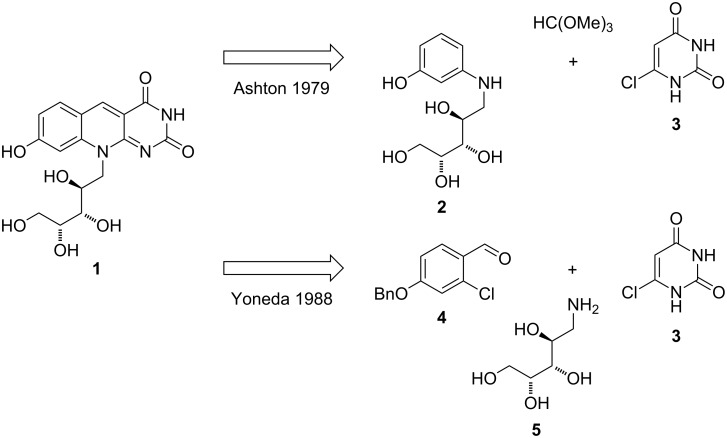
Target structure of this synthetic study and two previous approaches.

Its biosynthetic precursor, 7,8-didemethyl-8-hydroxy-5-deazariboflavin (**1**, Scheme 1) without a phosphodiester extension is called FO and came into our focus of interest because it was for instance suggested to function as an antenna pigment in several cryptochrome proteins [4]. One or more avian cryptochromes in turn [4–7] are very likely to be the primary magnetoreceptor molecules which enable migratory birds to perceive the compass direction of the Earth’s magnetic field through a light-dependent, spin-based, radical-pair mechanism in the birds’ eyes [8–11]. A major challenge slowing down progress towards understanding the exact magnetoreceptive properties of avian cryptochromes is that, so far, avian cryptochromes have been very difficult to express recombinantly with their co-factors (most likely a FAD and a FO (**1**) or a FAD and a 5,10-methenyltetrahydrofolate (MTHF) still attached [4,12–13]). Consequently, no crystal structures of a functional avian cryptochrome are known at present. While FAD and MTHF are commercially available, FO (**1**) is not. However, this compound is potentially needed in order to reconstitute expressed apo-cryptochrome proteins to their functional state. Therefore, finding an efficient and reliable way to synthesize compound FO (**1**) is important.

Up to the year 2015 the preparation of compound FO (**1**) was reported in the literature twice, both utilized commercially available chlorouracil **3** as C-ring synthon. The first report by Ashton et al. [14–15] used *N*-(D-ribityl)-3-hydroxyaniline (**2**) as A-ring fragment. The second report by Yoneda et al. [16–18] employed 4-benzyloxy-2-chlorobenzaldehyde (**4**) as building block for the A-ring. Both syntheses suffer from several drawbacks; these are the limited stability of intermediate products (e.g., oxidation of compound **2**) and low yields together with low chemoselectivities. Major disadvantage in our hands turned out to be actually the high polarity and low solubility of intermediate products, which makes purification rather tedious. For example, the preparation of amine **5** by reduction of the oxime and its conversion with chlorouracil **3** turned out to be impossible when following a literature protocol [19]. We therefore decided to develop a robust synthesis of a protected derivative of amine **5** which could follow standard carbohydrate chemistry and might then allow purification and characterization of intermediate products. While we were following the synthetic approach by Yoneda et al. with a protected ribose derivative, Foss and co-workers were obviously facing the same preparative problem. To our surprise and delight (that we were not the only group struggling with the reports of Ashton and Yoneda), they have published very recently a synthesis of compound **1** by a modified route [20] and achieve 34% yield over six steps starting from D-ribose, 3-aminophenol and barbituric acid. Nevertheless, we would like to communicate herein our synthetic approach to compound FO (**1**), which finally turned out to reliably yield the final product with high purity.

## Results and Discussion

### Synthesis of aldehyde **9**

**Scheme 2:**
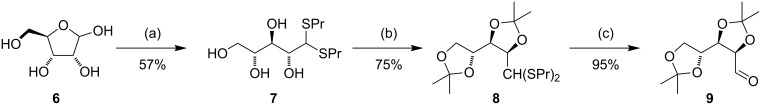
Preparation of isopropylidene protected ribose **9** along a new, modified route. Reagents and conditions: (a) 2 equiv *n-*PrSH, conc. HCl/H_2_O, 0 °C, 1.5 h; (b) 20 equiv 2,2-dimethoxypropane, 0.1 equiv *p*-TosOH·H_2_O, acetone, 2 h, 23 °C; (c) 3 equiv I_2_, 5.5 equiv NaHCO_3_, acetone/H_2_O (10:1), 20 h, 23 °C.

We envisioned the reduction of an oxime to be the most convenient access to the doubly isopropylidene protected amine **10** [21–22]. Therefore, we prepared diisopropylidene ribose **9** according to a literature sequence of dithioacetalization, acetonide formation and dithioacetal deprotection [23–25]. In particular, we have chosen the conversion of D-ribose (**6**) with *n*-propylthiol (Scheme 2), since the product **7** (57%) was crystalline [26]. Diacetonide formation (product **8**, 75%) proceeded as reported for the di(ethylthio) congener [24]. Finally, the dithioacetyl was cleaved while the isopropylidene moieties were retained, but not with mercury salts, as in original publications for the preparation of aldehyde **9** [27], but with the use of iodine (95% yield of product **9**), as recommended by Ohlsson et al*.* [28–29].

### Heterocyclic synthesis

**Scheme 3:**
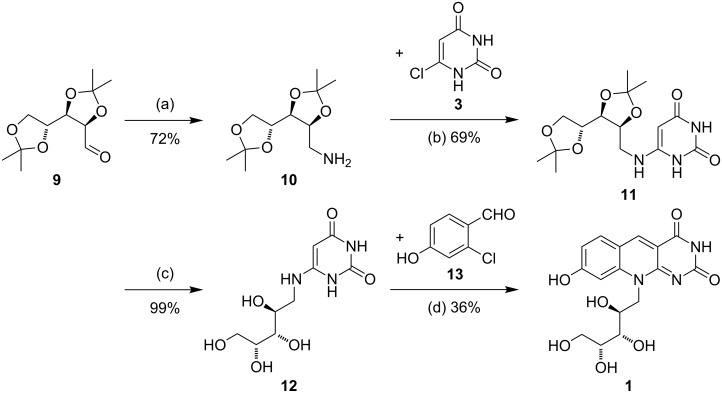
Final four steps of the synthesis. Reagents and conditions: (a) 1. 4.5 equiv NH_2_OH·HCl, 4 equiv NaHCO_3_, EtOH/H_2_O (10:1), 23 °C, 16 h; 2. 5 equiv LiAlH_4_, THF, 67 °C, 4 h; (b) 1.2 equiv amine **10**, 1.0 equiv chlorouracil **3**, 2.0 equiv NEt_3_, EtOH, sealed reaction tube, 150 °C, 1.5 h (c) TFA/H_2_O, 23 °C, 4 h; (d) 1 equiv aldehyde **13**, 1.2 equiv NEt_3_, EtOH, 150 °C, 3 h.

From the aldehyde **9** the corresponding oxime was formed under buffered conditions [30] and the crude material was directly submitted to reduction with LiAlH_4_ [31] to furnish amine **10** as a practically pure compound (72%) (Scheme 3). The conversion of amine **10** with chlorouracil **3** proceeded with NEt_3_ as base in EtOH at elevated temperature. Now, the key advantage of using amine **10** with isopropylidene-protected hydroxy functions came into the game, because product **11** could be purified by column chromatography. We first investigated the conversion of this material with aldehyde **13** to furnish the isopropylidene-protected annulation product. However, subsequent deprotection gave compound **1** containing significant impurities. If compound **11** is first deprotected (product **12** was obtained in 99% yield without impurities) and then converted with aldehyde **13**, the final target compound **1** could be crystallized from EtOH and washed with EtOH to furnish a pure, yellow crystalline material in 36% yield.

## Conclusion

We report on the new synthetic route to 7,8-didemethyl-8-hydroxy-5-deazariboflavin (**1**), which is the biosynthetic precursor compound to cofactor F420. In comparison with older routes to this target compound, the key innovation of our procedures is the introduction of two isopropylidene protective groups into the ribose side chain, which allowed the chromatographic purification of key intermediate product **11**. The sequence started with the preparation of literature-known bis-isopropylidene protected D-ribose **9**, which required a transitional protection of the aldehyde function as dithioacetal. With two deviations from the literature protocols (use of propyl- instead of ethylthiol and dithioacetal deprotection with iodine instead of mercury compounds) we have improved the synthesis of aldehyde **9**. The latter compound was then transformed to the protected ribitylamine **10** via the oxime, which was reduced with LiAlH_4_. Primary amine **10** was then converted with chlorouracil **3**, which was actually the key innovation of our new route, because the product **11** could be purified by column chromatography prior to deprotection to compound **12**. The insufficient purity of compound **12** was the major drawback of one of the previously known syntheses. Conversion of aminouracil **12** to the target compound **1** proceeded straightforwardly.

## Experimental

### 1-Amino-1-deoxy-2,3:4,5-bis-*O*-(isopropylidene)-D-ribitol (**10**)

**Formation of the oxime:** A solution of NaHCO_3_ (1.55 g, 18.4 mmol) and NH_2_OH·HCl (1.44 g, 20.7 mmol) in EtOH/H_2_O (10:1) was stirred for 30 min at ambient temperature. A solution of aldehyde **9** (1.06 g, 4.61 mmol) in EtOH (9 mL) was then added, and the resulting mixture stirred for further 16 h at ambient temperature. About half of the solvent was evaporated, the residue extracted with Et_2_O (3 × 20 mL) and the combined organic layers were dried (MgSO_4_) and evaporated after filtration to yield the crude oxime (985 mg, 4.00 mmol, 87%; mixture of *E*/*Z* isomers, ratio ca. 3:1), which was submitted to reduction without further purification. HRMS (ESI^+^): calcd for C_11_H_19_NNaO_5_ 268.1161, found 268.1157 [M + Na]^+^.

**Reduction to the amine 10:** A solution of the above reported oxime (985 mg, 4.00 mmol) in abs. THF (anhydrous, 20 mL) was dropwise added to a cooled (ice/water bath) suspension of LiAlH_4_ (760 mg, 20.0 mmol) in abs. THF (20 mL). The reaction mixture was heated for 4 h under reflux, then cooled with an ice/water bath and treated with MgSO_4_·7 H_2_O (20 g). The suspension was stirred for 1 h at ambient temperature, then filtered through a short pad of MgSO_4_ and the residue washed with MTBE (100 mL). The solvent was removed in vacuum to give amine **10** (771 mg, 3.33 mmol, 83%) as colorless oil; [α]^20^_D_ = −40 (CH_2_Cl_2_, 1 g/L); ^1^H NMR (500 MHz, CDCl_3_) δ 1.32 (s, 6H), 1.38 (s, 3H), 1.39 (s, 3H), 2.74 (brs, 2H), 2.90 (dd, *J* = 13.2 Hz, *J* = 7.7 Hz, 1H), 3.03 (dd, *J* = 13.2 Hz, *J* = 5.4 Hz, 1H), 3.89 (dd, *J* = 8.1 Hz, *J* = 5.1 Hz, 1H), 3.98 (dd, *J* = 9.1 Hz, *J* = 5.7 Hz, 1H), 4.03–4.11 (m, 2H), 4.19 (dt, *J* = 7.6 Hz, *J* = 5.5 Hz, 1H) ppm; ^13^C{^1^H} NMR (125 MHz, CDCl_3_) δ 25.42 (CH_3_), 25.43 (CH_3_), 26.7 (CH_3_), 28.1 (CH_3_), 41.2 (CH_2_), 68.0 (CH_2_), 73.2 (CH), 78.1 (CH), 79.2 (CH), 108.5 (C), 109.8 (C) ppm; IR (ATR): 3385 (w), 2986 (m), 2961 (m), 2935 (m), 2875 (w), 1571 (w), 1558 (w), 1481 (w), 1456 (m), 1400 (w), 1246 (s), 1213 (s), 1156 (s), 1061 (s), 980 (m), 894 (w), 845 (s), 791 (w), 754 (m) cm^−1^; HRMS (ESI^+^): calcd for C_11_H_22_NO_4_, 232.1543; found, 232.1539 [M + H^+^].

### 1-Deoxy-1-[(1,2,3,6-tetrahydro-2,6-dioxopyrimidin-4 yl)amino]-2,3:4,5-bis-*O*-(isopropylidene)-D-ribitol (**11**)

A mixture of chlorouracil **3** (218 mg, 1.49 mmol), ribitylamine **10** (413 mg, 1.79 mmol), NEt_3_ (0.41 mL, 2.98 mmol) and EtOH (3 mL) was heated in a closed and sealed reaction vial at 150 °C for 1.5 h. After cooling to ambient temperature the mixture was concentrated in vacuum and the residue purified by column chromatography (SiO_2_, MeOH/CH_2_Cl_2_ 1:20, *R*_f_ 0.06) to give title compound **11** (350 mg, 1.03 mmol, 69%) as a colorless solid; mp. 234–236 °C; [α]^20^_D_ = –35 (MeOH, 1 g/L); ^1^H NMR (500 MHz, CDCl_3_) δ 1.33 (s, 6H), 1.39 (s, 3H), 1.41 (s, 3H), 3.32–3.36 (m, 1H), 3.53 (dd, *J* = 13.9 Hz, *J* = 3.5 Hz, 1H), 3.84–3.91 (m, 1H), 4.02–4.16 (m, 3H), 4.41 (ddd, *J* = 9.0 Hz, *J* = 5.9 Hz, *J* = 3.6 Hz, 1H), 4.78 (s, 1H) 5.94 (s, 1H), 8.99 (brs, 1H), 10.40 (brs, 1H) ppm; ^13^C{^1^H} NMR (125 MHz, CDCl_3_) δ 25.28 (CH_3_), 25.30 (CH_3_), 26.7 (CH_3_), 27.8 (CH_3_), 42.2 (CH_2_), 69.0 (CH_2_), 73.0 (CH), 74.0 (CH), 75.0 (CH), 77.9 (CH), 109.5 (C), 111.1 (C), 152.3 (C), 154.9 (C), 165.9 (C) ppm; IR (ATR): 3360 (w), 3138 (w), 2987 (w), 2934 (w), 2881 (w), 1714 (s), 1626 (s), 1455 (m), 1381 (m), 1281 (m), 1212 (m), 1155 (m), 1069 (s), 976 (w), 838 (s), 783 (s), 629 (m) cm^−1^; HRMS (ESI^+^): calcd for C_15_H_23_N_3_NaO_6_, 364.1479; found, 364.1480 [M + Na^+^].

### 1-Deoxy-1-[(2,6-dioxo-1,2,3,6-tetrahydro-4-pyrimidinyl)amino]-D-ribitol (**12**)

A solution of compound **11** (245 mg, 0.72 mmol) in TFA/H_2_O (2.2 mL, 10:1) was stirred for 4 h at 23 °C. Subsequently, all volatile materials were removed in vacuum, the residue was suspended in Et_2_O (3 mL) and this suspension heated to reflux for 30 min. After cooling to ambient temperature, the precipitate was collected on a glass frit, washed with EtOH (1 mL) and dried under vacuum to give the deprotected ribitol **12** (185 mg, 0.71 mmol, 99%) as a colorless solid; mp. 183–185 °C. [α]^20^_D_ = –16.7 (DMSO, 1 g/L); ^1^H NMR (500 MHz, DMSO-*d*_6_) δ 3.01 (ddd, *J* = 12.5 Hz, *J* = 8.0 Hz, *J* = 4.6 Hz, 1H), 3.17 (ddd, *J* = 12.8 Hz, *J* = 5.6 Hz, *J* = 3.2 Hz, 1H), 3.37–3.41 (m, 2H), 3.48–3.51 (m, 1H), 3.57 (dd, *J* = 11.0 Hz, *J* = 3.3 Hz, 1H), 3.70–3.73 (m, 1H), 4.42 (s, 1H), 6.18 (s, 1H), 9.95 (brs, 1H), 10.15 (brs, 1H) ppm, signals for the four OH protons are not resolved (broad singlet from 4.5–5.0 ppm); ^13^C{^1^H} NMR (125 MHz, DMSO-*d*_6_) δ 43.9 (CH_2_), 63.2 (CH_2_), 69.5 (CH), 72.5 (CH), 72.8 (CH), 73.0 (CH), 150.8 (C), 154.4 (C), 164.5 (C) ppm; IR (ATR): 3296 (br m), 3144 (m), 3133 (m), 2980 (m), 2945 (m), 2889 (m), 1732 (m), 1709 (s), 1628 (s), 1603 (s), 1556 (m), 1483 (w), 1462 (w), 1345 (w), 1203 (w), 1048 (m), 1024 (m), 1000 (m) cm^−1^. HRMS (ESI^+^): calcd for C_9_H_16_N_3_O_6_, 262.1039; found, 262.1040 [M + H^+^].

### 1-Deoxy-1-(8-hydroxy-2,4-dioxo-2,3,4,10-tetrahydropyrimido[4,5-*b*]quinoline-10-yl)-D-ribitol (**1**)

A mixture of aldehyde **13** (90 mg, 0.57 mmol), aminouracil **12** (150 mg, 0.57 mmol), NEt_3_ (0.10 mL, 0.68 mmol) and EtOH (3 mL) was heated in a closed reaction vial at 150 °C for 3 h. After cooling to ambient temperature, the yellow precipitate was collected by filtration and washed with EtOH (3 mL) to give deazariboflavin **1** (75 mg, 0.21 mmol, 36%) as yellow solid, mp 240–250 °C (decomposition). [α]^20^_D_ = –6.7 (DMSO, 1 g/L); ^1^H NMR (500 MHz, CDCl_3_) δ 3.44–3.66 (m, 4H), 4.20–4.25 (m, 1H), 4.48 (brs, 1H), 4.64–4.83 (m, 3H), 4.90–4.98 (m, 1H), 5.09–5.15 (m, 1H), 7.02 (dd, *J* = 8.7 Hz, *J* = 1.6 Hz, 1H), 7.39 (s, 1H), 8.01 (d, *J* = 8.8 Hz, 1H), 8.87 (s, 1H), 10.96 (s, 1H), 11.24 (brs, 1H) ppm; ^13^C{^1^H} NMR (125 MHz, CDCl_3_) δ 47.9 (CH_2_), 63.4 (CH_2_), 69.6 (CH), 72.8 (CH), 73.9 (CH), 102.2 (CH), 110.8 (C), 115.3 (C), 115.4 (CH), 133.8 (CH), 141.5 (CH), 144.1 (C), 156.6 (C), 158.2 (C), 162.5 (C), 164.3 (C) ppm; IR (ATR): 3124 (br m), 3015 (br w), 2803 (br m), 1699 (m), 1645 (m), 1590 (s), 1570 (s), 1513 (s), 1473 (s), 1392 (m), 1376 (m), 1345 (m), 1255 (m), 1233 (m), 1218 (m), 1149 (m), 1063 (m), 1036 (m), 909 (w), 854 (w), 818 (w), 793 (w), 767 (w), 722 (w), 691 (w) cm^−1^; HRMS (ESI^+^): calcd for C_16_H_18_N_3_O_7_, 364.1139; found, 364.1152 [M + H^+^]; UV–vis (DMSO, *c* = 0.11 g/L): λ_max_ (lg ε) 411 (4.06), 455 nm (4.19); fluorescence (DMSO): λ_em_ = 482 (λ_irr_ = 412 or 456 nm), 441 nm (λ_irr_ = 412 nm).

## Supporting Information

File 1 General experimental methods, procedures and analytical data for the preparation of aldehyde **9** and copies of NMR spectra of all reported compounds.
