# Supplementary material for: A robust synthesis of 7,8-didemethyl-8-hydroxy-5-deazariboflavin
Source: Beilstein J Org Chem. 2016 May 6;12:912–7. doi: 10.3762/bjoc.12.89 (PMC4901869; doi:10.3762/bjoc.12.89)

Supporting Information

for

A robust synthesis of 7,8-didemethyl-8-hydroxy-5-deazariboflavin

Matthias Bender[1], Henrik Mouritsen[2,3] and Jens Christoffers*[1]

Address: [1]Institut für Chemie, Universität Oldenburg, Carl von Ossietzky-Str. 9–11, D-26129 Oldenburg, Germany, [2]Institut für Biologie und Umweltwissenschaften, Universität Oldenburg, D-26111 Oldenburg, Germany and [3]Centre for Neurosensory Sciences, University of Oldenburg, D-26111 Oldenburg, Germany

Email: Jens Christoffers - jens.christoffers@uni-oldenburg.de

*Corresponding author

General experimental methods, procedures and analytical data for the preparation of aldehyde 9 and copies of NMR spectra of all reported compounds

General Methods. Preparative column chromatography was carried out using Merck SiO_2 (0.035–0.070 mm, type 60 A) with hexane, *tert*-butyl methyl ether (MTBE), CH_2Cl_2 and MeOH as eluents. TLC was performed on Merck aluminum plates coated with SiO_2 F_{254}. ^{1}H and ^{13}CNMR spectra were recorded on a Bruker Avance DRX 500 instrument. Multiplicities of carbon signals were determined with DEPT experiments. MS and HRMS spectra were obtained with a Waters Micromass Q-TOF Premier (ESI) spectrometer. IR spectra were recorded on a Bruker Tensor 27 spectrometer equipped with a "GoldenGate" diamond ATR unit. Optical rotations were determined with a with a Perkin Elmer polarimeter 343. The UV–vis spectrum was recorded with a Shimadzu UV-1800 spectrometer, the fluorescence spectrum with a Shimadzu RF-5301PC spectrometer.

D-Ribosedi(propylthio)acetal (7): Propanethiol (1.4 ml, 27 mmol) was added at ambient temperature to a well stirred solution of D-ribose (**6**, 2.00 g, 13.3 mmol) in concentrated hydrochloric acid (2 ml) and the mixture was stirred for further 5 min. The reaction mixture was then cooled with an ice/water bath and stirring was continued for 1.5 h at this temperature. Water (10 ml) was added and the mixture was allowed to stand for 1 h with continued cooling (ice/water bath). The colorless residue was collected on a glass filter frit and was washed with chilled water (20 ml) and hexane (50 ml) to give mercaptal **7** (2.15 g, 7.56 mmol, 57%) as a colorless solid, mp. 82°C. $[\alpha]^{20}_{D}$ = –11.7° (CH_2Cl_2, 0.5 g/l). ^{1}H-NMR (500 MHz, $CDCl_3$): δ = 1.01 (t, *J* = 7.3 Hz, 3 H), 1.02 (t, *J* = 7.3 Hz, 3 H), 1.62–1.70 (m, 4 H), 2.47 (t, *J* = 5.9 Hz, 1 H), 2.63–2.76 (m, 4 H), 2.91 (d, *J* = 5.6 Hz, 1 H), 3.24 (d, *J* = 4.4 Hz, 1 H), 3.28 (d, *J* = 3.1 Hz, 1 H), 3.88–3.83 (m, 3 H), 3.89–3.98 (m, 2 H), 4.19 (d, *J* = 4.0 Hz, 1 H) ppm. ^{13}C{^{1}H}-NMR (125 MHz, $CDCl_3$): δ = 13.51 (CH_3), 13.56 (CH_3), 22.77 (CH_2), 22.93 (CH_2), 33.63 (CH_2), 33.79 (CH_2), 55.59 (CH), 63.26 (CH_2), 72.64 (CH), 73.19 (CH), 74.69 (CH) ppm. IR (ATR): 3395 (s), 3330 (s), 3265 (s), 2962 (m), 2929 (m), 2905 (m), 2871 (m), 1416 (w), 1452 (w), 1412 (w), 1395 (w), 1377 (w), 1327 (w), 1280 (w), 1231 (w), 1231 (w), 1197 (w), 1084 (m), 1041 (m), 1038 (w), 966 (m), 900 (m), 780 (w), 737 (w), 720 (w), 693 (w) cm^{-1}. HRMS (ESI, pos. mode): calcd. 307.1008 (for $C_{11}H_{24}NaO_4S_2$); found 307.1017 [M + Na^+]. $C_{11}H_{24}O_4S_2$ (284.44).

2,3:4,5-Bis-*O*-(isopropylidene)-D-ribosedi(propylthio)acetal (8): A solution of mercaptal **7** (6.45 g, 22.1 mmol), dimethoxypropane (54 ml, 0.44 mol) and *p*-TosOH · H_2O (419 mg, 2.20 mmol) in acetone (110 ml) was stirred for 2 h at ambient temperature. The mixture was then diluted with aqueous Na_2CO_3-solution (5%, 100 ml) and extracted with CH_2Cl_2 (3 x 100 ml). The organic layer was dried ($MgSO_4$) and after filtration, the solvent removed under vacuum. The residue was purified by column chromatography (SiO_2, hexane/MTBE 10:1, R_f = 0.30) to yield the title compound **8** (6.03 g, 16.5 mmol, 75%) as a colorless oil. $[\alpha]^{20}_{D}$ = –93.1° (CH_2Cl_2, 0.7 g/l). ^{1}H-NMR (500 MHz, $CDCl_3$): δ = 0.99 (t, *J* = 7.2 Hz, 3 H), 1.00 (t, *J* = 7.2 Hz, 3 H), 1.33 (s, 6 H), 1.40 (s, 3 H), 1.47 (s, 3 H), 1.57–1.69 (m, 4 H), 2.58–2.77 (m, 4 H), 3.89 (ddd, *J* = 8.5 Hz, *J* = 5.7 Hz, *J* = 1.4 Hz, 1 H), 4.15–4.06 (m, 2 H), 4.23 (dd, *J* = 4.2 Hz, *J* = 1.1 Hz, 1 H), 4.52 (ddd, *J* = 6.1 Hz, *J* = 4.2 Hz, *J* = 1.2 Hz, 1 H), 4.61 (dtd, *J* = 9.2 Hz, *J* = 5.9 Hz, *J* = 1.2 Hz, 1 H) ppm. ^{13}C{^{1}H}-NMR (125 MHz, $CDCl_3$): δ = 13.65 (CH_3), 13.75

(CH_3), 22.42 (CH_2), 22.72 (CH_2), 24.71 (CH_3), 25.40 (CH_3), 26.54 (CH_3), 26.76 (CH_3), 32.72 (CH_2), 33.27 (CH_2), 50.84 (CH), 68.24 (CH_2), 73.16 (CH), 79.00 (CH), 81.32 (CH), 109.11 (C), 109.66 (C) ppm. IR (ATR): 2984 (w), 2961 (w), 2961 (w), 2933 (w), 1456 (w), 1379 (m), 1370 (m), 1250 (m), 1210 (m), 1158 (m), 1061 (s), 985 (w), 968 (w), 936 (w), 887 (w), 849 (w), 795 (w), 710 (w) cm^{-1}. HRMS (ESI, pos. mode): calcd. 387.1634 (for $C_{17}H_{32}NaO_4S_2$), found 387.1638 [M + Na^+]. $C_{17}H_{32}O_4S_2$ (364.56).

2,3:4,5-Bis-*O*-(isopropylidene)-D-ribose (9): $NaHCO_3$ (2.30 g, 27.1 mmol) and iodine (3.4 g, 13 mmol) were successively added to a cooled (ice/water bath) solution of thioacetal **8** (2.2 g, 6.0 mmol) in a mixture of acetone/water (66 ml, 10:1). The reaction mixture was stirred for 16 h at ambient temperature. A second portion of $NaHCO_3$ (0.50 g, 6.0 mmol) and iodine (1.5 g, 5.0 mmol) was added. After stirring at ambient temperature for 4 h the mixture was treated with aqueous $Na_2S_2O_3$-solution (30%, 200 ml) and extracted with EtOAc (3 x 100 ml). The organic layer was dried ($MgSO_4$) and the solvent was removed under vacuum. The aldehyde **9** was obtained without further purification (1.32 g, 5.73 mmol, 95%) as colorless oil. $[\alpha]^{20}_D$ = –5.8° (CH_2Cl_2, 0.4 g/l). ^1H-NMR (500 MHz, $CDCl_3$): δ = 1.30 (s, 3 H), 1.37 (s, 3 H), 1.40 (s, 3 H), 1.53 (s, 3 H), 3.87–3.93 (m, 1 H), 4.06–4.13 (m, 2 H), 4.30 (t, *J* = 7.1 Hz, 1 H), 4.60 (d, *J* = 6.6 Hz, 1 H), 9.71 (s, 1 H) ppm. $^{13}C\{^1H\}$-NMR (125 MHz, $CDCl_3$): δ = 25.52 (CH_3), 25.85 (CH_3), 27.11 (CH_3), 27.76 (CH_3), 67.92 (CH_2), 73.98 (CH), 79.19 (CH), 82.19 (CH), 110.56 (C), 111.68 (C), 198.05 (CH) ppm. IR (ATR): 2987 (m), 2937 (w), 2833 (w), 1737 (m), 1457 (w), 1372 (m), 1245 (m), 1212 (s), 1155 (m), 1064 (s), 922 (w), 841 (s), 797 (w) cm^{-1}. HRMS (ESI, pos. mode): calcd. 231.1227 (for $C_{11}H_{19}O_5$), found 231.1228 [M + H^+]. $C_{11}H_{18}O_5$ (230.26).

Copies of ^{1}H and ^{13}C{^{1}H} NMR spectra of all reported compounds:

D-Ribosedi(propylthio)acetal (7):

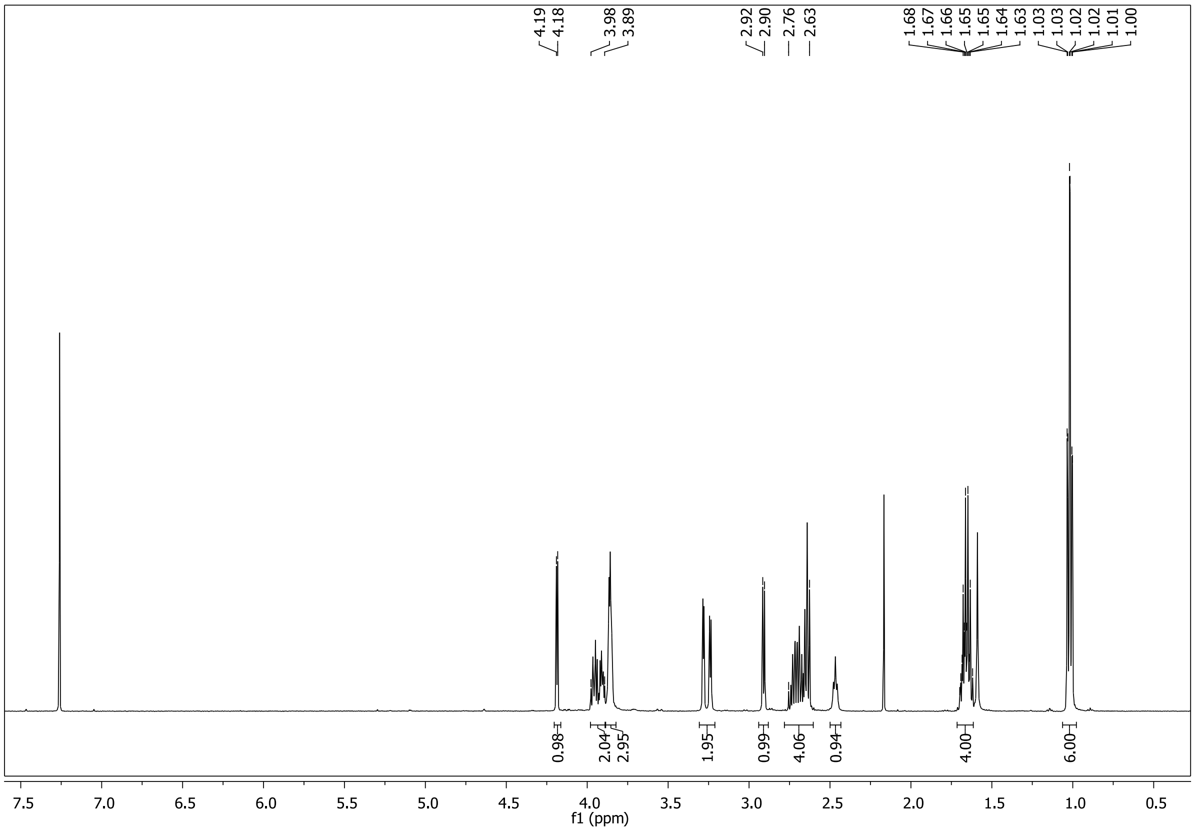

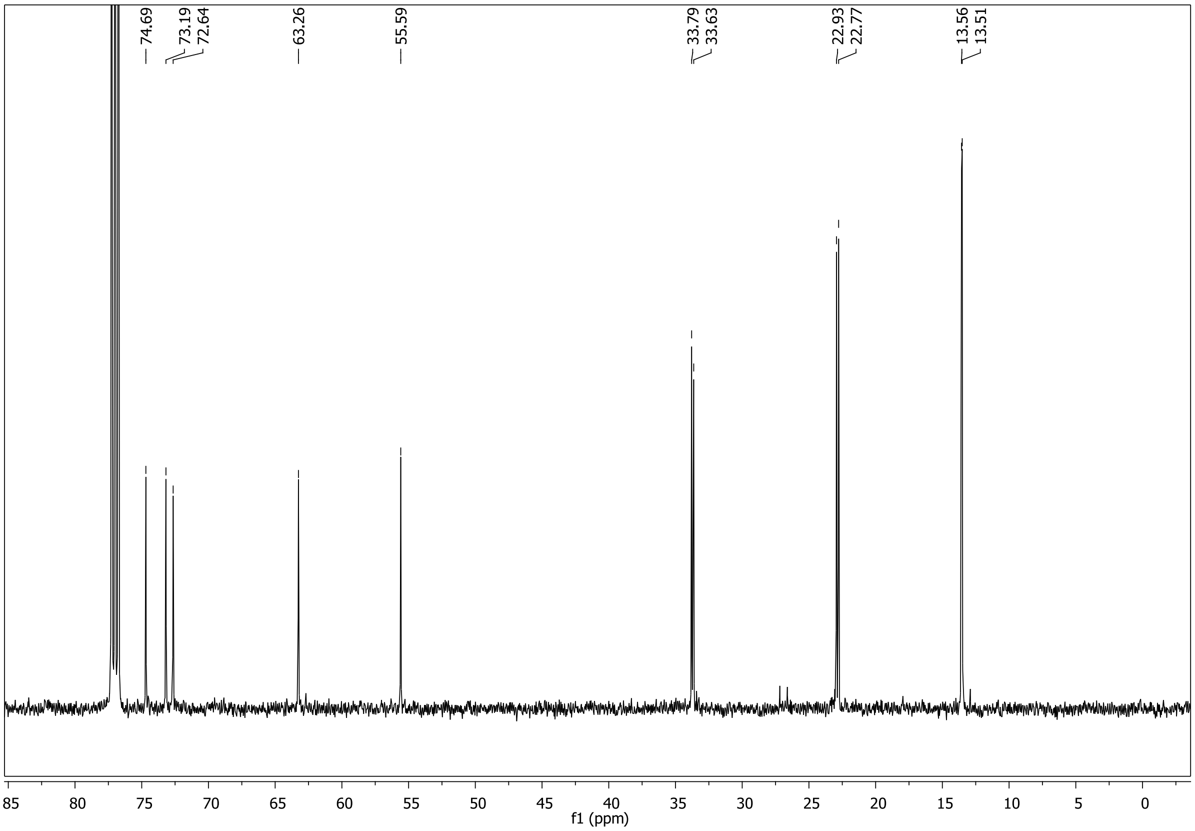

2,3:4,5-Bis-*O*-(isopropylidene)-D-ribosedi(propylthio)acetal (8):

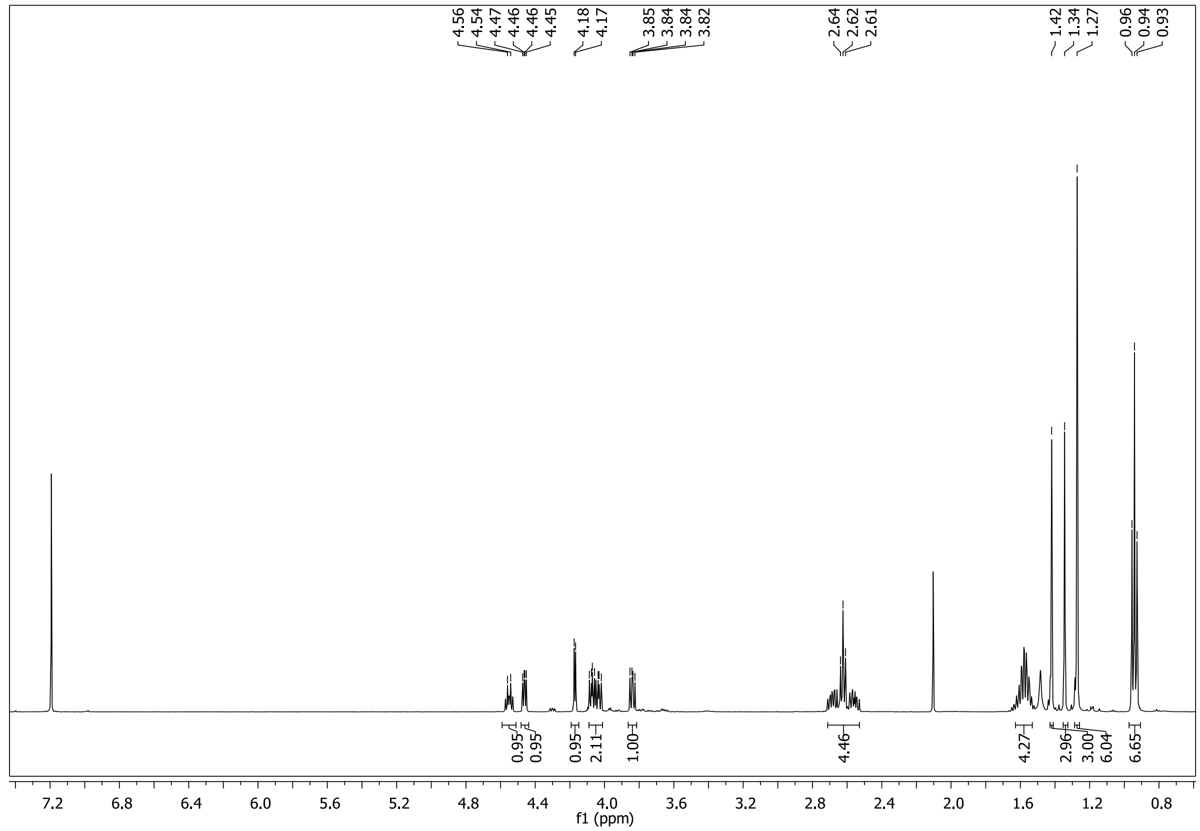

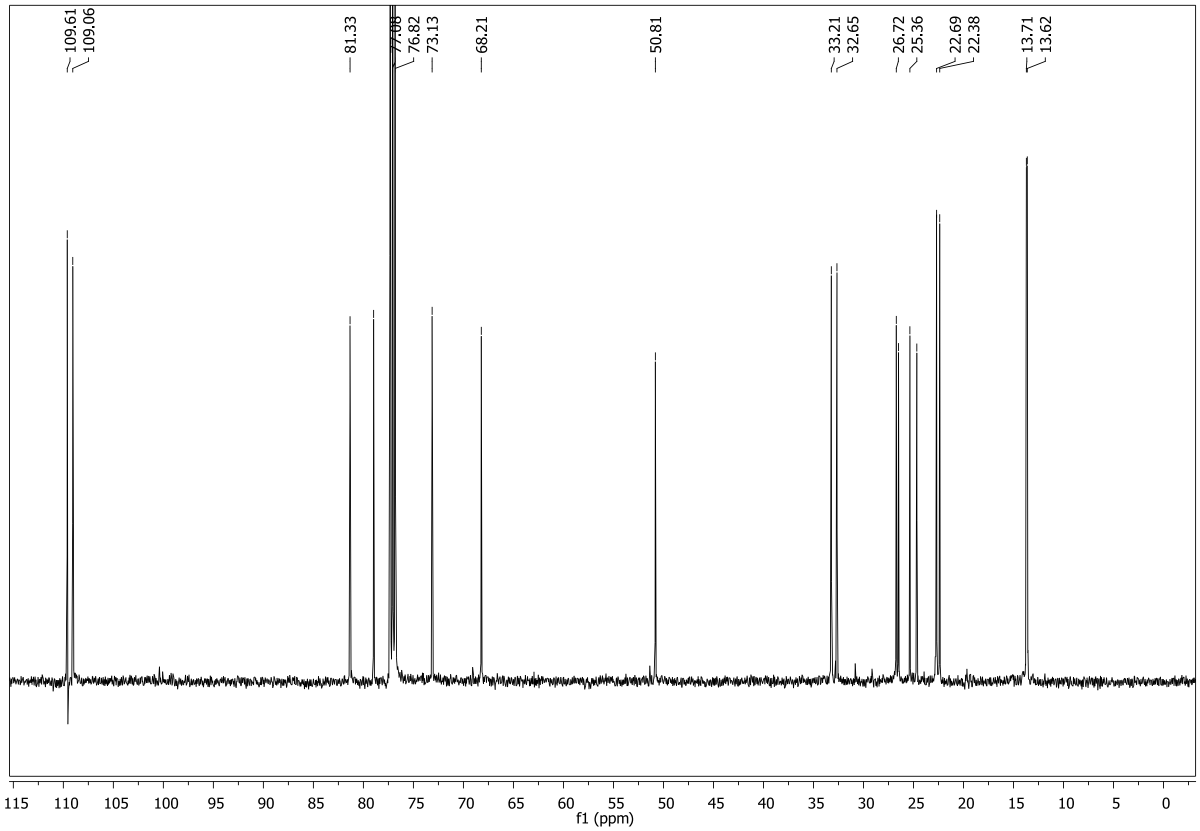

2,3:4,5-Bis-*O*-(isopropylidene)-D-ribose (9):

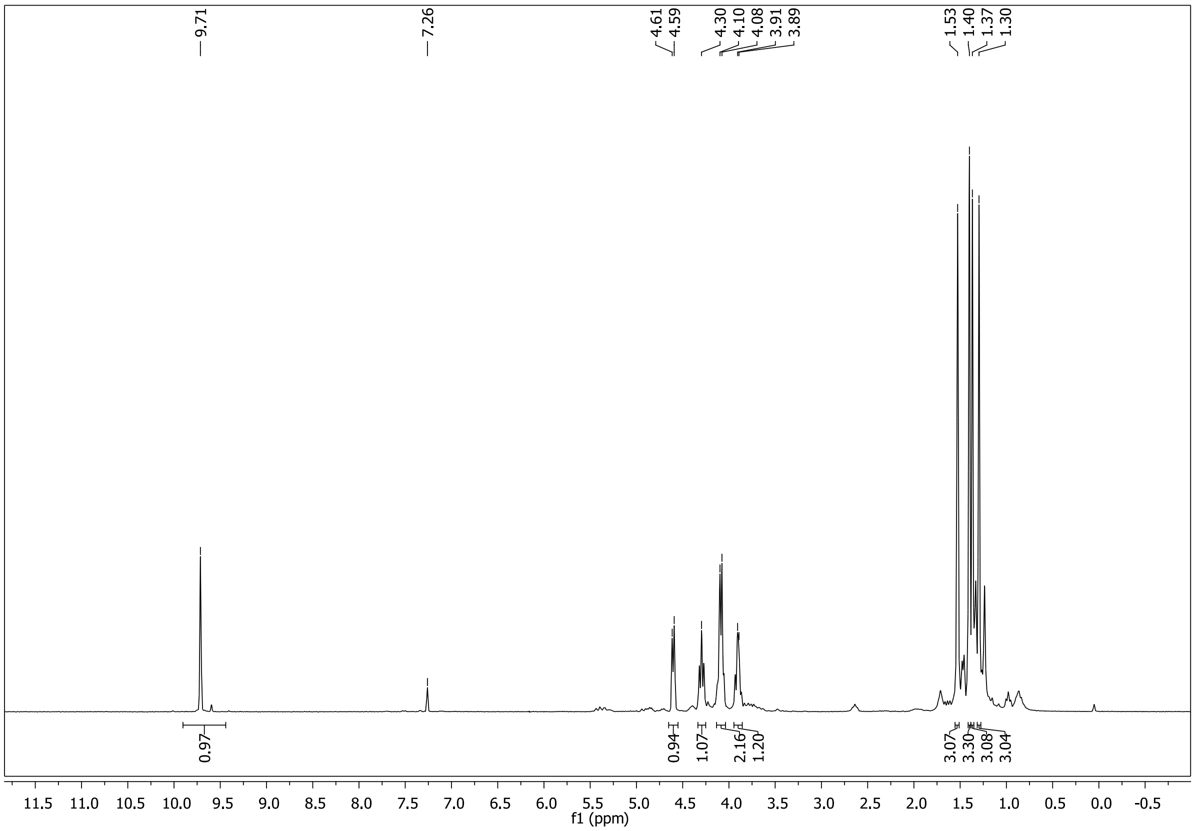

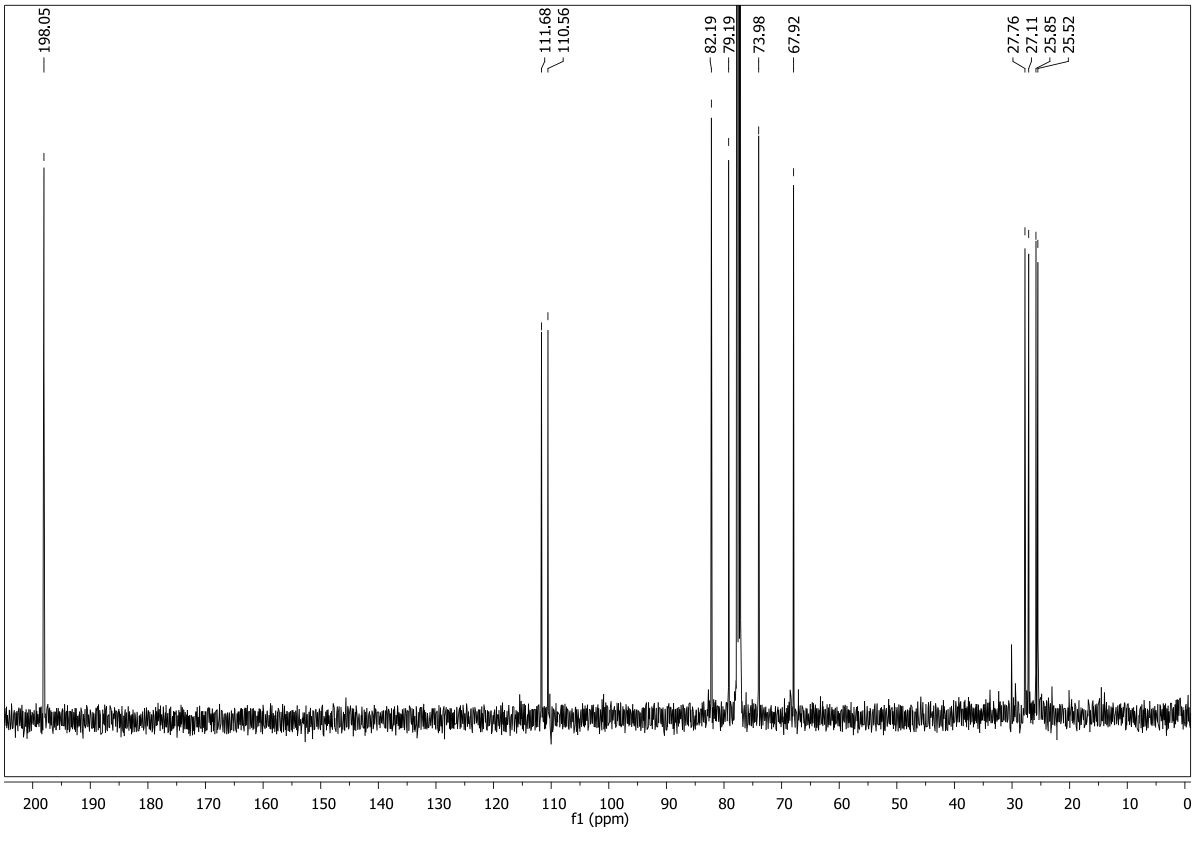

1-Amino-1-deoxy-2,3:4,5-bis-*O*-(isopropylidene)-D-ribitol (10):

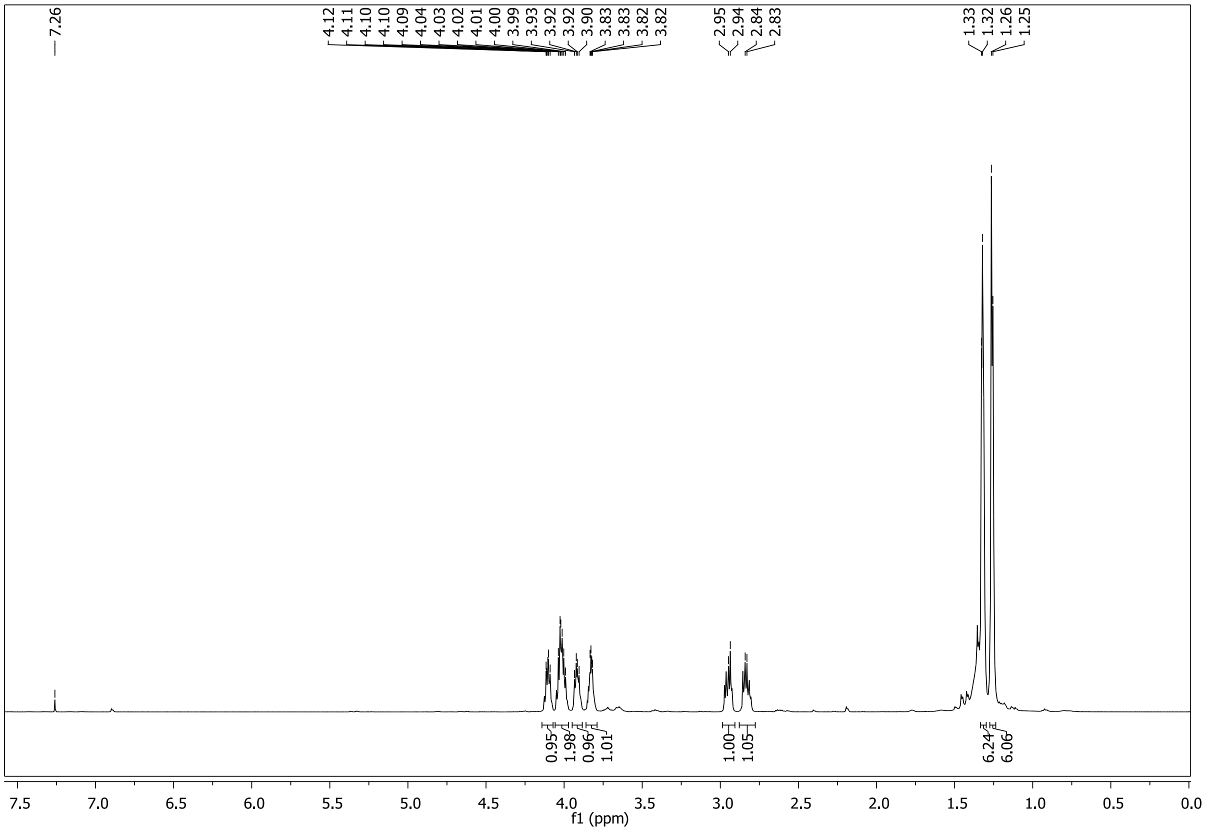

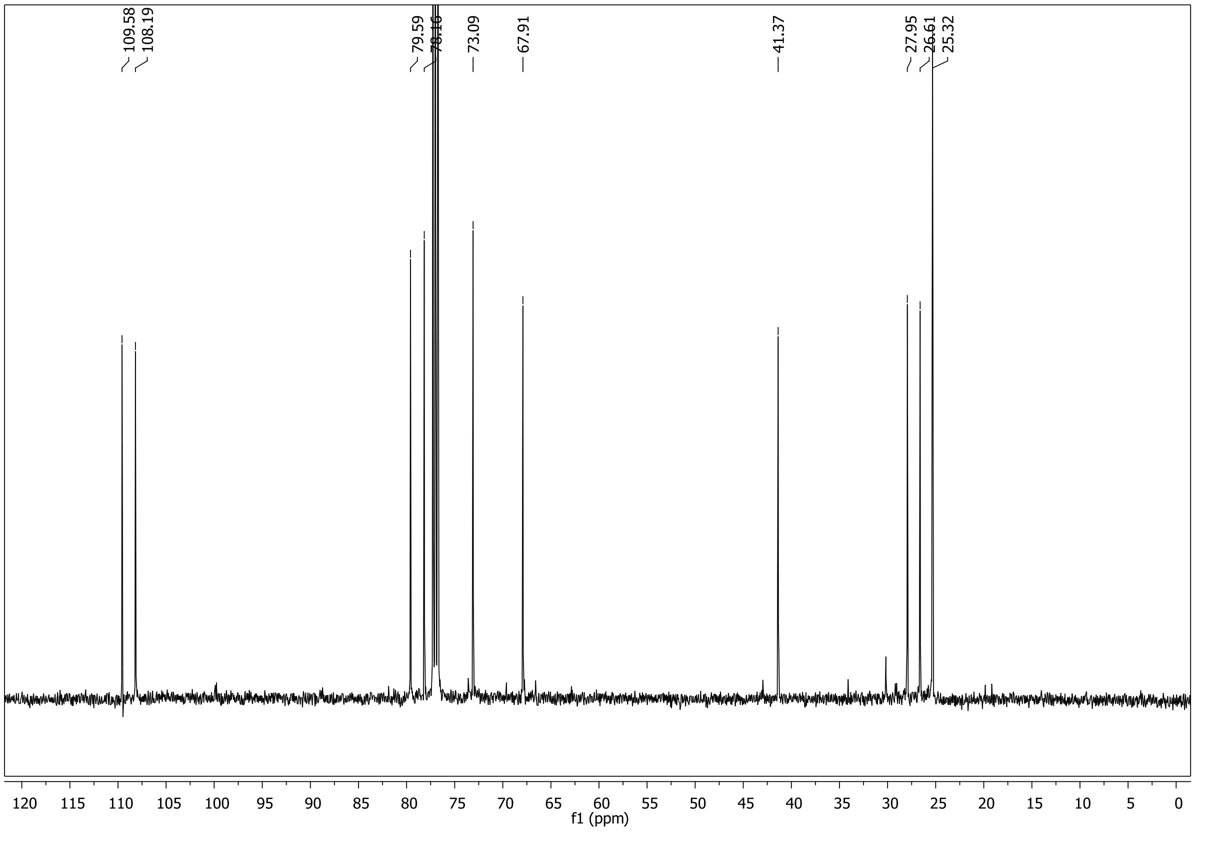

1-Deoxy-1-[(1,2,3,6-tetrahydro-2,6-dioxo-4-pyrimidinyl)amino]-2,3:4,5-bis-*O*-(isopropylidene)-D-ribitol (11):

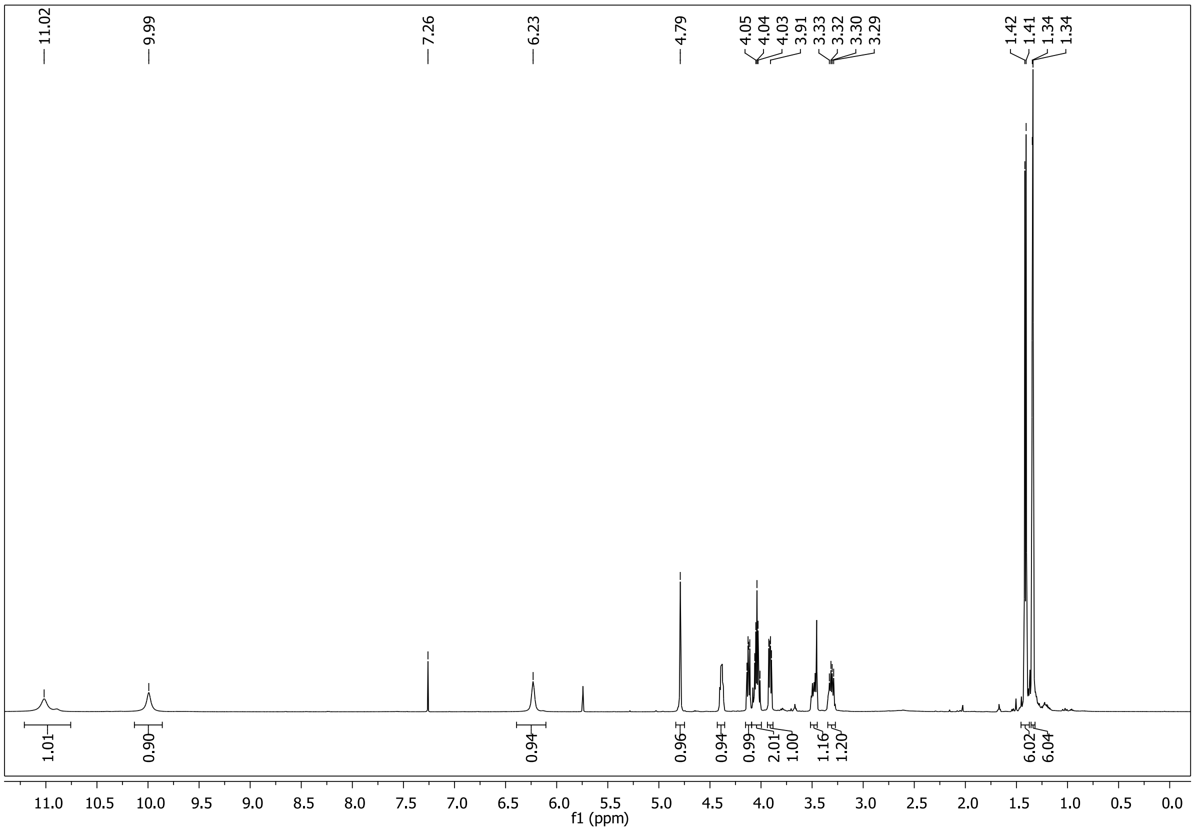

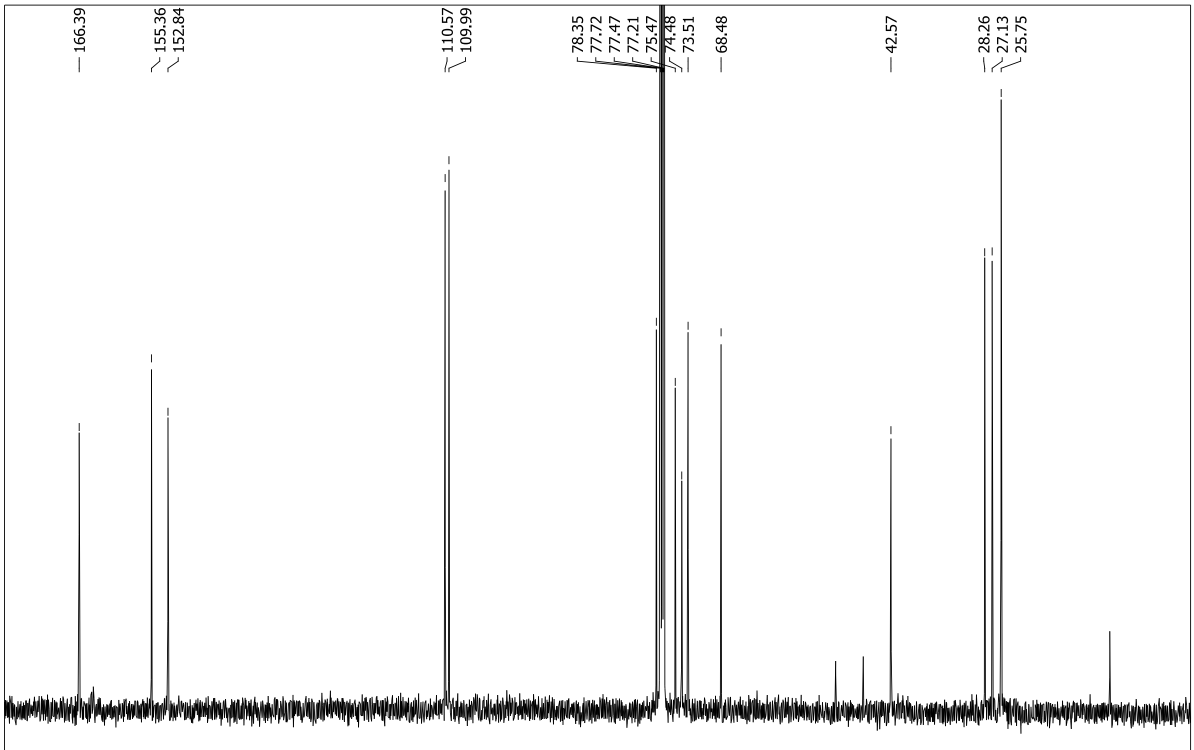

1-Deoxy-1-[(2,6-dioxo-1,2,3,6-tetrahydro-4-pyrimidinyl)amino]-D-ribitol (12):

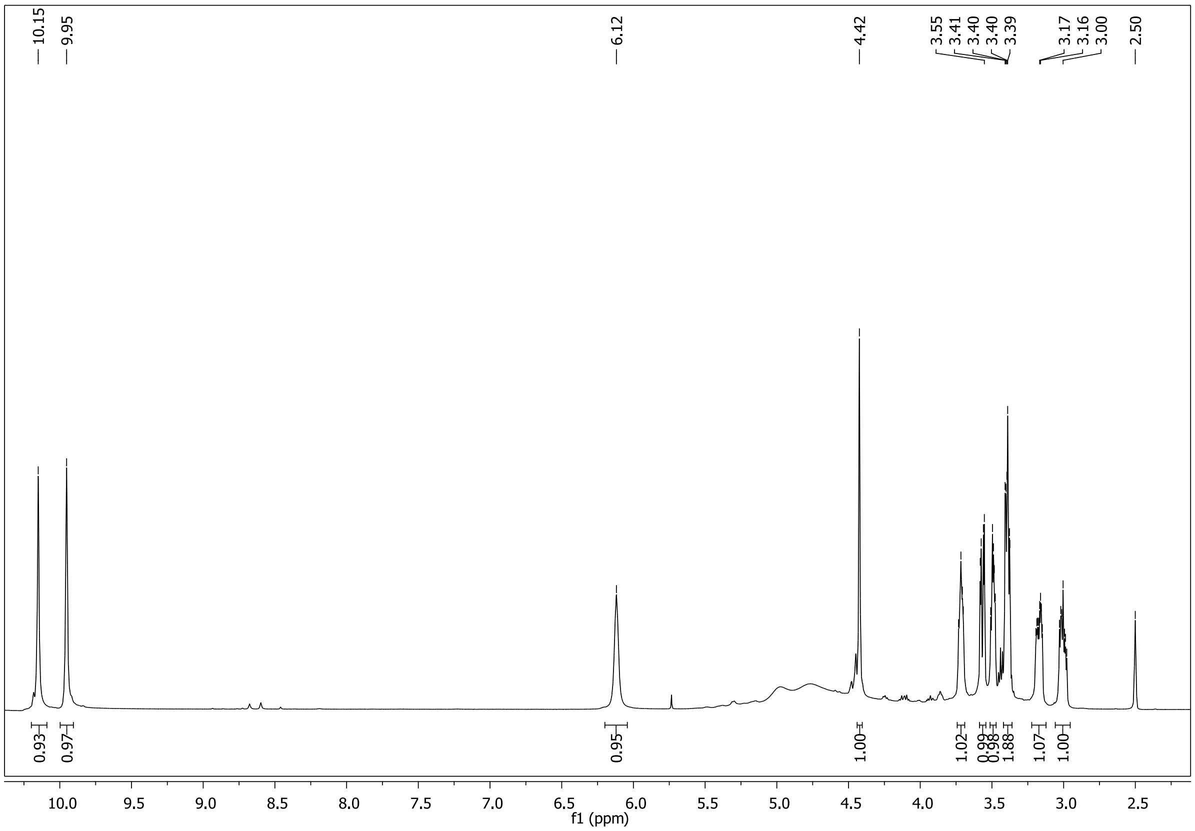

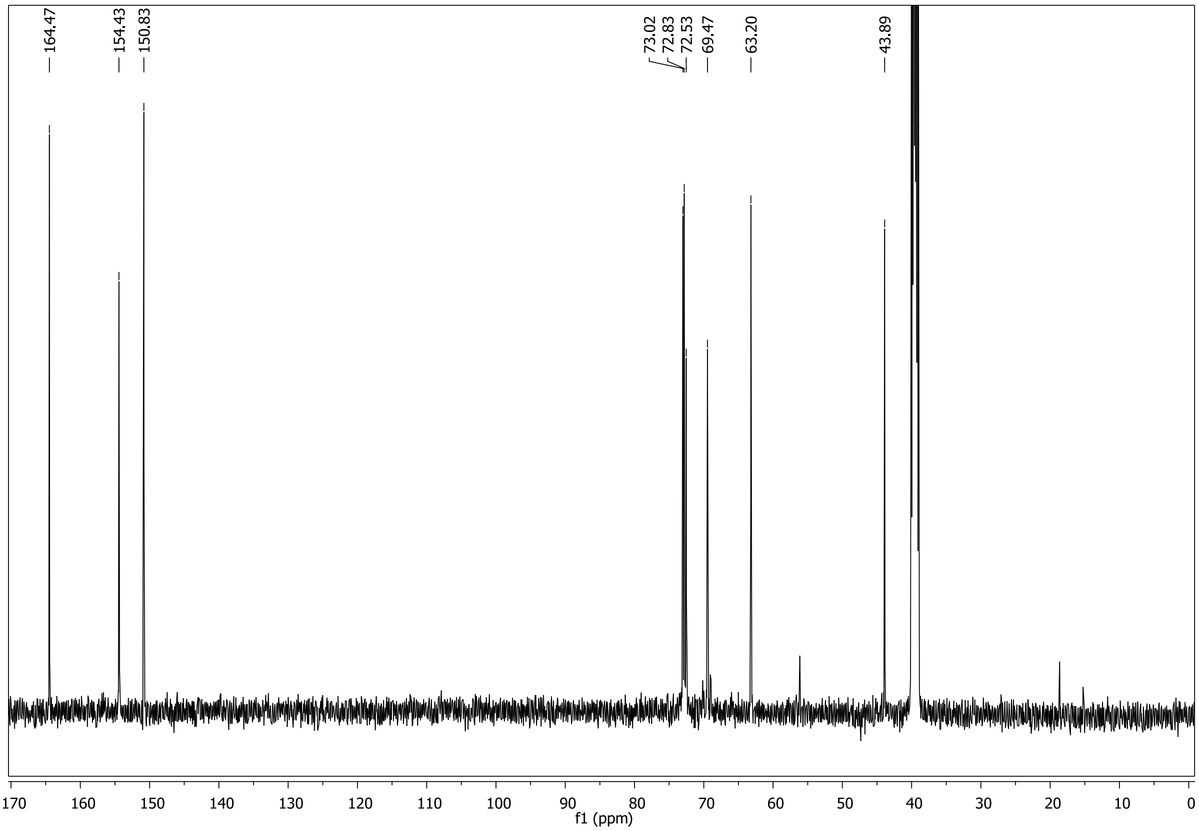

1-Deoxy-1-(8-hydroxy-2,4-dioxo-2,3,4,10-tetrahydropyrimido[4,5-b]quinoline-10-yl)-D-ribitol (1):

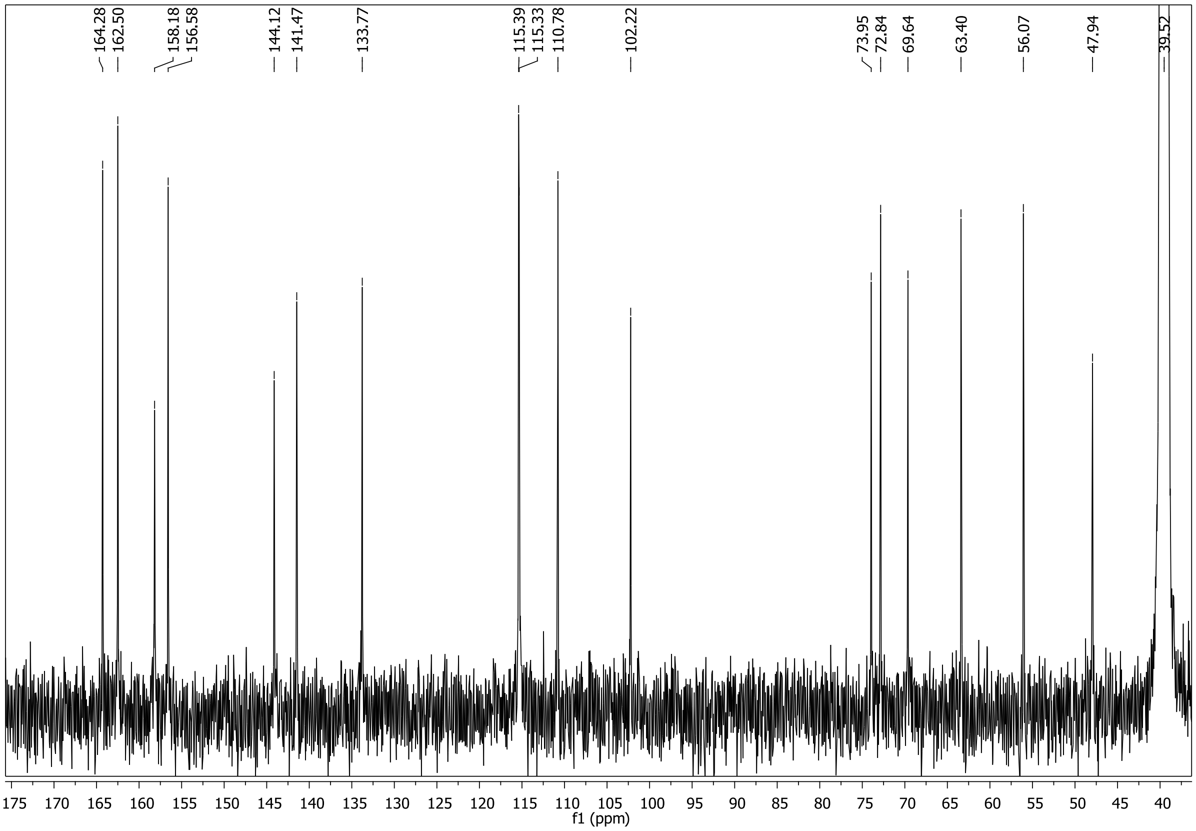

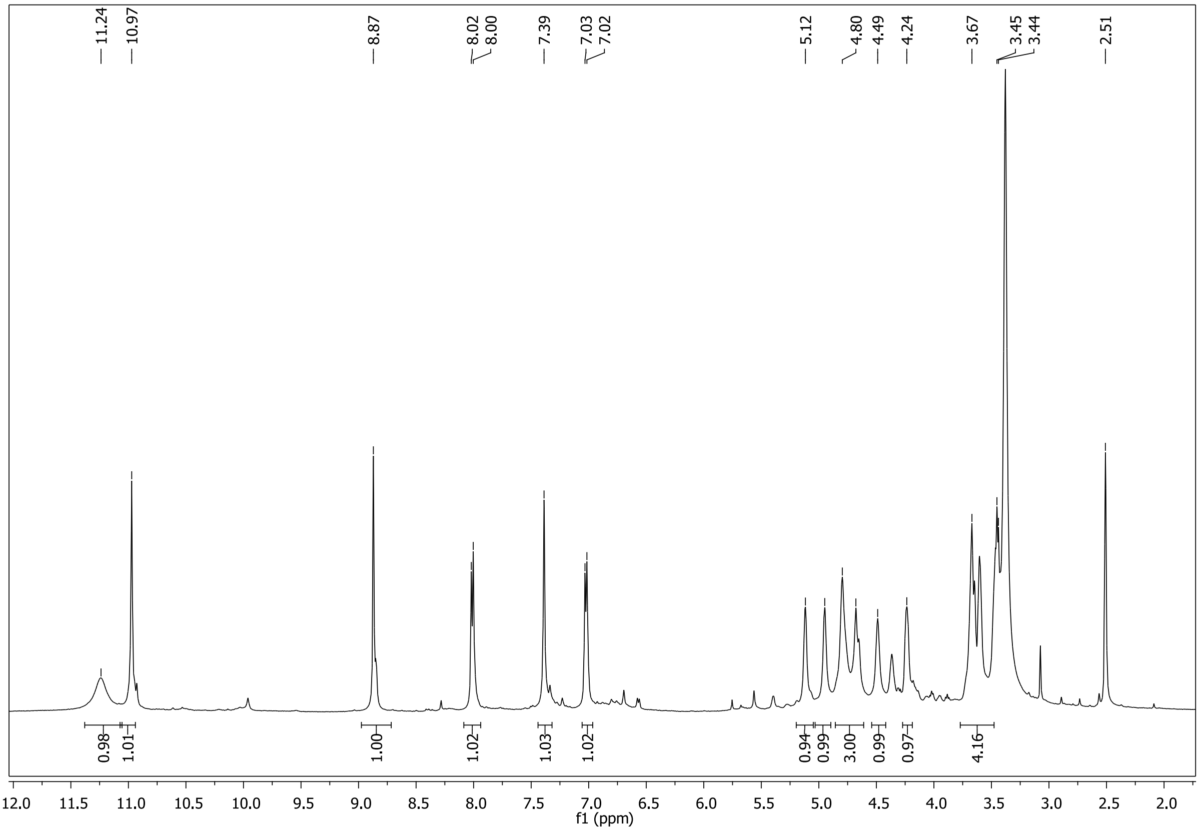

Supplement: File 1 — General experimental methods, procedures and analytical data for the preparation of aldehyde 9 and copies of NMR spectra of all reported compounds. [file Beilstein_J_Org_Chem-12-912-s001.pdf]
